# An Improved Kernel Entropy Component Analysis for Damage Detection Under Environmental and Operational Variations

**DOI:** 10.3390/s25051332

**Published:** 2025-02-21

**Authors:** Shuigen Hu, Jian Yang, Jiezhong Huang, Dongsheng Li, Cheng Li

**Affiliations:** 1 Anhui Provincial International Joint Research Center of Data Diagnosis and Smart Maintenance on Bridge Structures, Chuzhou 239099, China; hushuigen@chzu.edu.cn; 2 Department of Civil and Intelligent Construction Engineering, Shantou University, Shantou 515063, China; 3 Guangdong Engineering Center for Structure Safety and Health Monitoring, Shantou University, Shantou 515063, China; 4 Shantou Key Laboratory of Offshore Wind Energy, Shantou 515063, China; 5 Key Laboratory for Health and Safety of Bridge Structures, Wuhan 430034, China

**Keywords:** structural health monitoring, damage detection, environmental and operational variation, variational mode decomposition, principal component analysis, gaussian process regression

## Abstract

Environmental effects often trigger false alarms in vibration-based damage detection methods used for structural health monitoring (SHM). While conventional techniques like Principal Component Analysis (PCA) and cointegration have been somewhat effective in addressing this issue, challenges such as measurement noise, nonlinear behavior, and non-Gaussian data distribution continue to affect their performance. To address these limitations, a novel damage detection method combining Variational Mode Decomposition (VMD) and Dynamic Kernel Entropy Component Analysis (DKECA) is proposed. The proposed method initially uses the VMD technique to remove seasonal patterns and noise from the modal frequencies. Subsequently, a DKECA model is constructed based on a time-delay data matrix, and the principal components that maximize the Rényi entropy in the high-dimensional space are selected. Using these principal components, a damage detector developed from the $T^{2}$ statistic is used to determine damage indices for SHM. The effectiveness of the proposed method is verified through both a simulated 7-DOF model and real-world data from the Z24 bridge, with comparative studies highlighting its advantages over existing techniques.

## 1. Introduction

Structural damage detection plays a critical role in the safety management of civil engineering structures [1]. It facilitates the early identification of structural issues, leading to a significant reduction in maintenance costs. In recent years, vibration-based damage detection methods have gained significant attention. The principle behind these methods is that structural damage alters structural vibration properties, such as frequency and mode shapes. By analyzing changes in these properties, damage can be detected through an inverse problem-solving approach. However, in real-world applications, structural vibration properties are not solely influenced by damage; they are also affected by changing environmental and operational conditions (EOCs), such as temperature [2], humidity [3], traffic [4], and wind [5]. These environmental factors may have an equal or even greater impact on vibration properties than the damage itself, making it difficult to reliably detect structural damage. Therefore, it is crucial to account for environmental effects when conducting damage detection.

Until now, researchers have proposed several approaches to mitigate these environmental effects, and these have generally been classified into explicit and implicit methods [6]. In explicit methods, a regression model is used to establish the relationship between environmental variables and vibration properties, with the model’s residual serving as a damage indicator that is insensitive to EOCs. Common explicit methods include linear regression [7], polynomial regression [8], autoregressive models with inputs [9], Gaussian process regression [10], random forest [11], support vector machines [12], and neural networks [13]. The advantage of the explicit method is that it can quantify the impact of environmental factors (input) on dynamic characteristics (output), and the established model is easy to interpret. However, to establish an accurate input–output relationship, the potentially influential environment variables should be comprehensively considered. In practice, it may not be possible to measure all influential input variables for civil structures [14].

Implicit methods, on the other hand, do not require direct measurement of environmental variables and have become increasingly popular. The key idea is to treat environmental influences as hidden variables in the dynamic data, separating the damage-related effects from environmental effects by projecting the data into orthogonal subspaces. Common implicit methods include PCA [15], factor analysis [16], Gaussian mixture models [17], Mahalanobis distance [18], and the cointegration method [19]. However, most of these methods are based on linear mappings, which are only effective when there is a strong linear correlation between the vibration properties. In reality, environmental factors often have a nonlinear impact on structural vibration properties, which will cause a nonlinear correlation between monitored vibration properties. For example, nonlinear relationships between modal frequencies are observed in multiple real bridges during long-term monitoring, such as the Z24 bridge [9], Dowling Hall Footbridge [8], and the KW51 bridge [7]. In such scenarios, the effectiveness of traditional implicit damage detection methods is limited.

One approach to address the limitations of traditional implicit methods is to use piecewise linearization, which divides nonlinearly related data into multiple segments of linearly related data and then uses linear methods for removing environmental effects. For example, Yan et al. [20] introduced a local PCA method. This approach divides damage features into multiple linear regions and applies linear PCA separately within each region to remove environmental effects. Similarly, Zang et al. [21] proposed the subdomain PCA method, which builds multiple PCA models across distinct linear subdomains of nonlinear frequency data. More recently, Shi [22] and Huang [23] developed a regime-switching cointegration approach to mitigate the effects of environmental variations in SHM data with nonlinear relationships. This method transforms nonlinear damage features into piecewise linear ones based on a switching temperature and then employs the cointegration method to remove environmental effects on each linear dataset. However, the disadvantage of the piecewise linearization method is that it is only suitable for bilinear or multilinear data, and so its performance is limited to more complex nonlinear correlation data.

To handle more complex nonlinear problems, the kernel principal component analysis method is proposed. Kernel principal component analysis (KPCA) is a type of kernel-based machine learning method that first maps the original dataset into a high-dimensional space using a kernel trick in which the process variables are linearly correlated. Then, the standard PCA is performed in the high-dimensional space. Due to its ability to handle complex nonlinear data, KPCA is widely used in image processing [24], pattern recognition [25], and process monitoring [26]. Recently, the KPCA method has also been applied to the SHM field. For example, Reynders et al. [27] used KPCA to eliminate environmental and operational influences, and the misfit between the KPCA prediction and original data was used to detect damage occurrence. Bisheh et al. [28] proposed a novel damage detection method based on the variational mode decomposition technique and KPCA under the influences of environmental and operational changes, KPCA is performed on the constructed feature matrix to remove environmental effects and obtain damage-sensitive indices. Additionally, other studies [29,30] have explored similar KPCA-based approaches to improve damage detection in structural health monitoring.

Although previous studies have proved that the KPCA method can effectively process nonlinear data as well as remove nonlinear environmental effects, two major drawbacks were noticed. Firstly, this method is based on PCA, and PCA assumes a Gaussian distribution of the variables [31]. However, the data may deviate from the Gaussian distribution due to complex environmental effects, which may cause the principal components extracted by KPCA to not accurately reflect the characteristics of the data, which may produce inaccurate and misleading results. Secondly, long-term monitoring data show non-stationary dynamic changes under the influence of changing EOCs, and they have different time series correlations in the dynamic monitoring process. However, the traditional KPCA model is a static model, which ignores the correlation characteristics of the variables over time. Therefore, KPCA is not suitable for monitoring the dynamic process [32].

To address the limitations of traditional KPCA, a VMD-DKECA method is proposed for damage detection under varying EOCs. At the core of this approach is kernel entropy component analysis (KECA), which integrates kernel methods with entropy measures to analyze and decompose complex data patterns. KECA’s focus on entropy-based criteria enables it to effectively capture components that maximize the information content of the data, offering robustness against non-Gaussian characteristics and improving performance in feature extraction and dimensionality reduction [33]. To account for the time correlation of variables in dynamic monitoring processes, a time-lagged data extension is incorporated into KECA, forming a dynamic KECA model. This extension enhances the model’s ability to describe the dynamic properties of the processes. Additionally, to mitigate the challenges posed by seasonal environmental variations and measurement noise, VMD is employed as a preprocessing step to filter out these effects from the modal frequencies. This combined approach ensures more reliable and efficient damage detection. The VMD-DKECA method is tested on both a simulated 7-DOF example and a real bridge structure. A comparison study of PCA, KPCA, KECA, and DKPCA is investigated in terms of false positive rate and false negative rate. The results confirm that the proposed approach delivers superior performance in detecting damage.

The structure of this paper is as follows: Section 1 introduces the background theory of KECA. Section 2 provides an overview of the VMD method and outlines the VMD-DKECA process for damage detection. In Section 3, a numerical simulation using a 7-DOF spring-mass system is presented. Section 4 applies the proposed method to the Z24 Bridge benchmark study to validate its effectiveness. This paper concludes with a summary in Section 5.

## 2. Kernel Entropy Component Analysis (KECA)

KECA is a data-driven method used to extract features for process monitoring, especially in non-linear and high-dimensional systems. The core idea of KECA lies in the concept of entropy in information theory, which measures the uncertainty or disorder within a dataset. By applying kernel methods, which project data into a higher-dimensional space, KECA identifies components that capture the maximum entropy of the data, thus revealing the most informative features for monitoring [34].

Consider a dataset $X={\{x}_{1},x_{2},x_{3},\cdots ,x_{n}\}\in R^{d\times n}$ with a probability density distribution p(x), where *d* is the dimension of damage features and *n* represents the number of samplings. The Rényi entropy for this dataset is defined as follows:

(1) $$H\left(p\right)=-lg\int p^{2}\left(x\right)dx$$

Due to the monotonicity of the logarithmic function, the Rényi entropy is influenced by the function $V\left(p\right)=\int p^{2}(x)dx$. To estimate $V\left(p\right)$, the Parzen window probability density estimator is introduced:

(2) $$\hat{p}(x)=\frac{1}{n}\sum_{x_{t}\epsilon X}k_{\sigma }\left(x,x_{t}\right)$$

where $k_{\sigma }\left(x,x_{t}\right)$ is the Parzen window function centered at $x_{t}$; *σ* is the smoothing width. By using the sample mean to approximate $V\left(p\right)$, we obtain [34] the following:

(3) $$\begin{array}{c} \hat{V}\left(p\right)=\frac{1}{n}\sum_{x_{t}\epsilon X}\hat{p}\left(x_{t}\right)=\frac{1}{n}\sum_{x_{t}\epsilon X}\frac{1}{n}\sum_{x_{t}^{\prime }\epsilon X}k_{\sigma }\left(x_{t},x_{t}^{\prime }\right)=\frac{1}{n^{2}}1^{T}K1 \end{array}$$

Here, K is an n×n kernel matrix; 1 is a n×1 vector where each element equals one. From Equation (3), we can see that the Rényi entropy estimator fully relies on the kernel matrix elements. Furthermore, the Rényi entropy estimator can be expressed through the eigenvalues and eigenvectors of the kernel matrix, and is decomposed as follows:

(4) $$K=E\Lambda E^{T}$$

where E is a matrix containing eigenvectors $e_{1},e_{2},e_{3},\cdots ,e_{n}$, and Λ is a diagonal matrix containing eigenvalues $\lambda_{1},\lambda_{2},\lambda_{3},\cdots ,\lambda_{n}$. Based on the eigenvalues and eigenvectors of the kernel matrix, the estimated value of the Rényi entropy can be represented as [34] follows:

(5) $$\hat{V}(p)=\frac{1}{n^{2}}\sum_{i=1}^{n}{\left(\sqrt{\lambda_{i}}e_{i}^{T}1\right)}^{2}$$

This shows that certain eigenvalues and eigenvectors contribute more significantly to the entropy estimate than others.

Let $\phi :\;x_{i}\to \phi (x_{i})$ denote a nonlinear map from input space to feature space, and let $\Phi =\left[\phi \left(x_{1}\right),\;\phi \left(x_{2}\right),\;\cdots ,\;\phi \left(x_{n}\right)\right]$. In the KPCA method, the principal axes are chosen based on the size of eigenvalues, and the projection of Φ onto the *i*-th principal axes $u_{i}$ is defined as $u_{i}^{T}\Phi =\sqrt{\lambda_{i}}e_{i}^{T}$. Unlike the traditional KPCA, KECA chooses the principal axes relying on the joint contribution of eigenvalues and eigenvectors to Rényi entropy. Therefore, by selecting the first *m* eigenvalues and eigenvectors that most significantly contribute to the entropy value, we can obtain the transformed data using the KECA method [35]:

(6) $$S_{m}=U_{m}\Phi =\Lambda_{m}^{1/2}E_{m}^{T}:\;\underset{\lambda_{1},e_{1}\cdots \lambda_{n},e_{n}}{\min}\hat{V}\left(p\right)-{\hat{V}}_{m}(p)$$

where $S_{m}$ is the score matrix, $\Lambda_{m}$ is the diagonal matrix containing *m* eigenvalues sorted by their contribution to entropy, and $E_{m}$ is the corresponding eigenvector matrix.

For out-of-sample (testing) data, the transformation is given by [35] the following:

(7) $$S_{m}^{test}=U_{m}\Phi_{new}=\Lambda_{m}^{-1/2}E_{m}^{T}K_{test}^{T}$$

where $K_{new}=\Phi_{new}^{T}\Phi$. The KECA method has the following advantages compared to the KPCA method: (1) KECA selects the principal components based on their contribution to maximizing entropy, which directly relates to the information content, making it better for extracting the most informative features; (2) KPCA primarily focuses on maximizing variance in the projected feature space, which works well for Gaussian or near-Gaussian data distributions. In contrast, KECA goes beyond variance maximization by leveraging the concept of entropy, making it more suitable for data with complex, non-Gaussian distributions.

## 3. Damage Detection Based on VMD-DKECA

While the KECA method efficiently processes nonlinear and non-Gaussian data, it assumes temporal independence in regularly collected samples, overlooking the evolving characteristics of variables over time. In the long-term SHM process, however, monitoring variables typically exhibit dynamic changes due to varying environmental conditions, with different time-series correlations emerging throughout the monitoring process. Additionally, seasonal environmental factors and measurement noise can cause inconsistencies in the data, resulting in unreliable and inefficient damage detection [36]. To address these issues, an extended version of KECA, named VMD-DKECA, is developed in this paper. This approach is specifically designed to handle dynamic, nonlinear, and non-Gaussian monitoring data more effectively.

### 3.1. Variational Modal Decomposition

VMD is an adaptive and intrinsic signal processing algorithm used to decompose complex, nonlinear, and nonstationary signals into a series of local vibrational modes known as intrinsic mode functions (IMFs) [37]. The process of VMD can be considered as the construction and solution of a constrained variational problem, which can be expressed as follows [37]:

(8) $$\begin{array}{c} \underset{\left\{u_{k}\right\},\left\{\omega_{k}\right\}}{\min}\sum_{k=1}^{N}{\left\|\partial_{t}\left[\left(\delta \left(t\right)+\frac{j}{\pi t}\right)*u_{k}\left(t\right)\right]e^{-j\omega_{k}t}\right\|}_{2}^{2},\;s.t.\sum_{k=1}^{N}u_{k}\left(t\right)=f\left(t\right) \end{array}$$

where N is the number of the IMF, $u_{k}$ and $\omega_{k}$ are the *k*-th IMF and its center frequency, $f\left(t\right)$ is the original signal, $\delta \left(t\right)$ is the Dirac function, and ∗ is the convolution operation. $\partial_{t}$ is the gradient function of t. To solve the constrained variational problem, a quadratic penalty term α and the Lagrangian multiplier λ are introduced to render the variational problem unconstrained [37]:

(9) $$\begin{array}{c} \begin{array}{c} L\left({\{u}_{k}\},{\{\omega }_{k}\},\lambda \right)=\alpha \sum_{k=1}^{N}{\left\|\partial_{t}\left[\left(\delta \left(t\right)+\frac{j}{\pi t}\right)*u_{k}\left(t\right)\right]e^{-j\omega_{k}t}\right\|}_{2}^{2} \end{array}\\ +{\left\|f\left(t\right)-\sum_{k=1}^{N}u_{k}\left(t\right)\right\|}_{2}^{2}+\langle \lambda \left(t\right),f\left(t\right)-\sum_{k=1}^{N}u_{k}\left(t\right)\rangle \end{array}$$

where $\partial_{t}$ denotes the gradient operator, ∗ denotes the convolution operator, 〈 〉 denotes the inner product operator, ${\left\|\cdot \right\|}_{2}$ represents the 2-norm of a vector, and $\sum_{k=1}^{N}u_{k}\left(t\right)$ is understood as the summation over all modes. Equation (9) can be solved using the alternate direction method of multipliers (ADMM). Initially, the decomposition mode number is predetermined. Each mode $u_{k}^{1}$ in the Fourier domain, the corresponding center frequency $\omega_{k}^{1}$, and the Largrangian multiplier λ are initialized. Subsequently, the modes ${\hat{u}}_{k}$ and the center frequencies $\omega_{k}$ are updated using Equations (10) and (11), respectively [38].

(10) $${\hat{u}}_{k}^{\tau +1}\left(\omega \right)=\frac{\hat{f}\left(\omega \right)-\sum_{i<k}{\hat{u}}_{i}^{\tau +1}\left(\omega \right)-\sum_{i>k}{\hat{u}}_{i}^{\tau }\left(\omega \right)+\frac{{\hat{\lambda }}^{\tau }\left(\omega \right)}{2}}{1+2\alpha {\left(\omega -\omega_{k}^{\tau }\right)}^{2}}$$

(11) $$\omega_{k}^{\tau +1}=\frac{\int_{0}^{\infty }\omega {\left|{\hat{u}}_{k}^{\tau +1}\left(\omega \right)\right|}^{2}d\omega }{\int_{0}^{\infty }{\left|{\hat{u}}_{k}^{\tau +1}\left(\omega \right)\right|}^{2}d\omega }$$

where τ is the iteration number, f(ω), u(ω), and  λ(ω) denote the Fourier transform of f(t), u(t), and λ(t), respectively. Then, following the same dual ascent step in the ADMM algorithm, the Lagrangian multiplier is updated, which can be expressed as [39] follows:

(12) $${\hat{\lambda }}^{\tau +1}\left(\omega \right)={\hat{\lambda }}^{\tau }\left(\omega \right)+\beta (\hat{f}\left(\omega \right)-\sum_{k=1}^{N}{\hat{u}}_{k}^{\tau +1}\left(\omega \right))$$

The above iteration continues until convergence is achieved:

(13) $$\frac{\sum_{k=1}^{N}{\left\|{\hat{u}}_{k}^{\tau +1}-{\hat{u}}_{k}^{\tau }\right\|}_{2}^{2}}{\sum_{k=1}^{N}{\left\|{\hat{u}}_{k}^{\tau }\right\|}_{2}^{2}}<\epsilon$$

where the value ϵ is normally set as 10^−6^ and all IMFs can be recovered according to the above loop. In this paper, the VMD method is used to decompose the original frequency data into two IMFs. IMF1 corresponds to the nonstationary long-run pattern, while IMF2 corresponds to the short-run stationary seasonal pattern [40]. By removing the seasonal patterns (IMF2), the retained IMF1 is used to establish the DKECA model. The specific VMD settings used in this research are outlined below:

*k* = 2: This parameter represents the number of IMFs into which the original signal is decomposed. In this study, the signal is decomposed into two components: a short-term stationary seasonal pattern and a non-stationary long-term trend. Thus, *k* is set to 2.

α represents the quadratic penalty term, which aims to reduce the interference of Gaussian noise. The larger the value of *α*, the less noise there is in the decomposition process. In the wooden truss bridge example in this paper, its value is set to 100, and, in the Z24 bridge example, its value is set to 10.

ϵ represents the tolerance parameter that controls the convergence speed of the algorithm. When a smaller ϵ value is selected, the VMD algorithm takes longer to converge. This paper sets $\epsilon =10^{-7}$.

init represents the initialization center frequency of the IMF. It can be set to 0 (zero initialization), 1 (uniform initialization), or 2 (random initialization). Studies have shown that the initialization of the center frequency has little effect on the decomposition results. Therefore, init is set to 0.

τ is the time-step of the dual ascent that determines the updating of the Lagrangian multipliers (generally defined to have an exact reconstruction or to achieve denoising).

### 3.2. Dynamic Analysis of Monitoring Data

During long-term structural monitoring, the vibration properties of structures fluctuate dynamically due to changing environmental conditions. Measurement data often exhibit strong correlations influenced by common environmental factors. However, KECA does not account for the autocorrelation among variables, leading to less effective performance when applied to dynamic processes. To overcome this limitation, DKECA is introduced to capture the temporal dynamics of monitoring data, thereby enhancing the accuracy of damage detection.

In DKECA, the normalized original data matrix is denoted as $X={\{x}_{1},x_{2},\cdots ,x_{n}\},$ where $x_{t}$ represents the sample vector in the time *t*. To capture the underlying dynamic relationships between variables, a dynamic dataset consisting of the current sample $x_{t}$ and previous samples $x_{t},\;x_{t-1},\cdots ,\;x_{t-l}$ is used. This dataset includes data from both the current moment and the previous l time points. A time-lagged matrix $\tilde{X}\left(l\right)$ is then constructed to reflect these dynamic relationships:

(14) $$\begin{array}{c} \tilde{X}\left(l\right)=\left[\begin{array}{ccc} x_{t}^{T} & x_{t-1}^{T} & \begin{array}{cc} \cdots & x_{t-l}^{T} \end{array}\\ x_{t-1}^{T} & x_{t-2}^{T} & \begin{array}{cc} \cdots & x_{t-l-1}^{T} \end{array}\\ \begin{array}{c} \vdots \\ x_{t+l-n}^{T} \end{array} & \begin{array}{c} \vdots \\ x_{t+l-N-1}^{T} \end{array} & \begin{array}{c} \begin{array}{cc} \vdots & \vdots \end{array}\\ \begin{array}{cc} \cdots & x_{t-N}^{T} \end{array} \end{array} \end{array}\right] \end{array}$$

Here, l represents the time lag step length, and the DKECA model is established by time-lagged matrix $\tilde{X}\left(l\right)$. This time-lagged matrix extends the observation variables to include data from the previous *l* moments, effectively capturing time correlations near the sampling point. This approach proves to be effective in handling processes with dynamic behavior [41]. The KECA and DKECA methods share the following similarities: (1) Both methods utilize the kernel trick to map data into a higher-dimensional feature space, enabling the identification of linear separability and improving data structure handling, which is particularly effective for nonlinear relationships. (2) Both methods rely on entropy as a key concept, using it to quantify uncertainty and randomness in the data. The goal is to extract components that minimize entropy, thereby enhancing data representation.

The primary difference between KECA and DKECA lies in the incorporation of a dynamic component in DKECA. Unlike KECA, which processes static data, DKECA accounts for time-dependent changes and dynamic variations, making it particularly suitable for datasets with temporal or sequential dependencies.

### 3.3. Proposed VMD-DKECA Damage Detection Method

Environmental effects can be categorized into two types: (1) short-term, stationary seasonal patterns, and (2) non-stationary, long-term trends [36]. Traditional damage detection methods often struggle with noise resulting from variance changes in heteroscedastic data, especially from seasonal variations. To address this challenge, we propose the use of the VMD technique as a preprocessing step. VMD decomposes frequency signals into two IMFs. The first IMF represents a zero-centered frequency, while the second IMF captures cyclic patterns linked to seasonal noise. By removing the second IMF, we isolate the first IMF for damage detection, effectively reducing the influence of seasonal effects.

To tackle nonlinear environmental influences in non-stationary long-term trends, we introduce DKECA, which applies the kernel trick to map data to a higher-dimensional space, enabling the capture of complex, nonlinear relationships. This capability allows DKECA to identify intricate patterns in dynamic environments. Furthermore, DKECA utilizes entropy as a key feature in the extraction process. Entropy, a measure of uncertainty or disorder, is particularly effective in handling non-Gaussian data, which generally exhibit higher and more complex entropy compared to Gaussian data. By combining VMD and DKECA, our approach effectively separates seasonal environmental effects and manages nonlinear, non-Gaussian dynamic data. This method enhances the robustness of structural health monitoring systems, ensuring reliable damage detection under varying environmental conditions.

The proposed VMD-DKECA method for damage detection can be divided into three phases: (1) data pre-processing, (2) offline modeling, and (3) online detection. In the data pre-processing phase, the VMD method is used to denoise the signals and remove the seasonal patterns in them. In offline modeling, the training dataset in an undamaged state is used to build the DKECA model. Additionally, in the online detection phase, the test datasets are projected onto the KECA model and a $T^{2}$ statistics are calculated to determine whether the damage occurs or not. The specific steps of the proposed VMD-DKECA method are described below.

#### 3.3.1. Data Pre-Processing

Before performing the main damage detection procedure, VMD is employed as a data preprocessing tool to decompose the original frequency data into two IMFs. IMF1 corresponds to the nonstationary long-run pattern, while IMF2 corresponds to the short-run stationary seasonal pattern. By removing the seasonal pattern, the retained IMF1 is divided into two parts: training data and test data.

#### 3.3.2. Offline Modelling

Step 1: Based on (10), the time-lagged matrix $\tilde{X}$ of the normalized training data is constructed;

Step 2: Calculate the kernel matrix of the time-lagged matrix $\tilde{X}$, and calculate the entropy value of the data according to (7);

Step 3: Based on (10), select the eigenvalues and eigenvectors that contribute mainly to the entropy, and extract the nonlinear component $S_{m}$;

Step 4: Calculate the $T^{2}$ monitoring statistics by $T^{2}=S_{m}\Lambda^{-1}S_{m}^{T}$, and determine the control limits of the statistics chart via the kernel density method.

Online detection

Step 1: Normalize the test data samples and construct the time-lagged matrix ${\tilde{X}}_{test}$;

Step 2: Project ${\tilde{X}}_{test}$ into the feature space, and extract the nonlinear principal component $S_{m}^{test}$;

Step 3: Calculate the monitoring statistics $T^{2}$, and perform damage detection according to whether the statistics exceed the control limit.

**Figure 1:**
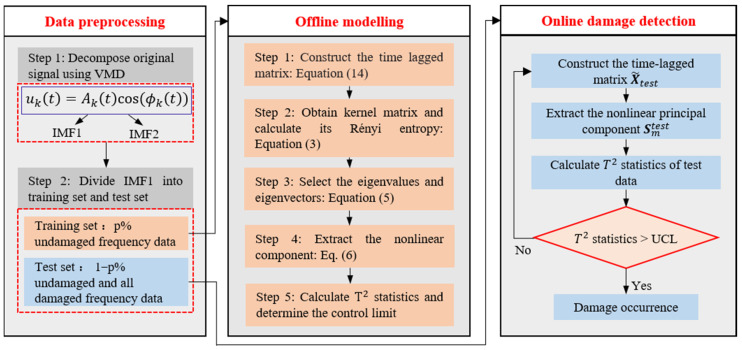
Procedure of the proposed VMD-DKECA damage detection method.

To provide a more comprehensive understanding of the algorithm’s stages, Figure 1 illustrates the flowchart of the proposed damage detection strategy.

## 4. Numerical Simulation: A 7-DOF Spring-Mass Model

**Figure 2:**
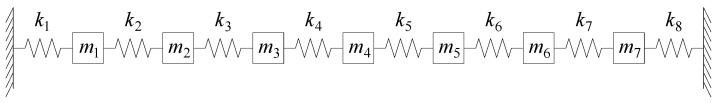
7-DOF spring–mass system.

**Figure 3:**
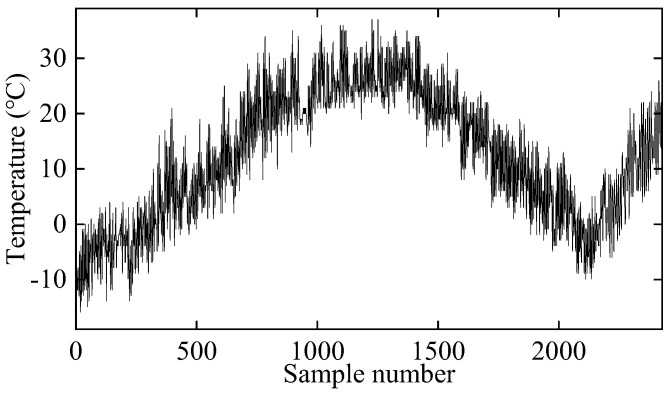
The variation of air temperature over time.

In this section, a numerical example featuring a seven-degree-of-freedom (7-DOF) system is employed to validate the proposed method’s effectiveness. The 7-DOF system, shown in Figure 2, comprises a chain structure anchored at both ends, with each lumped mass weighing 2 kg. To account for nonlinear environmental effects, the relationship between stiffness and temperature is modeled as follows [42]:

(15) $$k_{i}=\left\{\begin{array}{c} -0.15\times T+6,\;if\;T<0\\ -0.05\times T+6,\;ifT\geq 0 \end{array}\right.$$

for i=1,2,4,5,6,7,8, and

(16) $$k_{3}=\left\{\begin{array}{c} -0.1\times T+10,\;if\;T<0\\ -0.25\times T+10,\;if\;T\geq 0 \end{array}\right.$$

where $k_{i}$ denotes the stiffness of the *i*th spring, while T represents temperature. Temperature data from actual records in Beijing are utilized to simulate varying environmental conditions, encompassing 2189 sample points from 2013 and 239 sample points from March 2014, as depicted in Figure 3. Two distinct damage scenarios are examined: in the first scenario, $k_{2}$ decreases by 20% between sample points 2190 and 2310, while, in the second scenario, it decreases by 30% between sample points 2311 and 2428. Each stiffness coefficient is dependent on temperature, allowing for the determination of natural frequencies through the generalized eigenvalue problem $\left|M-\omega^{2}K(T)\right|=0$. Additionally, a small amount of 2% Gaussian white noise is added to simulate measurement errors.

**Figure 4:**
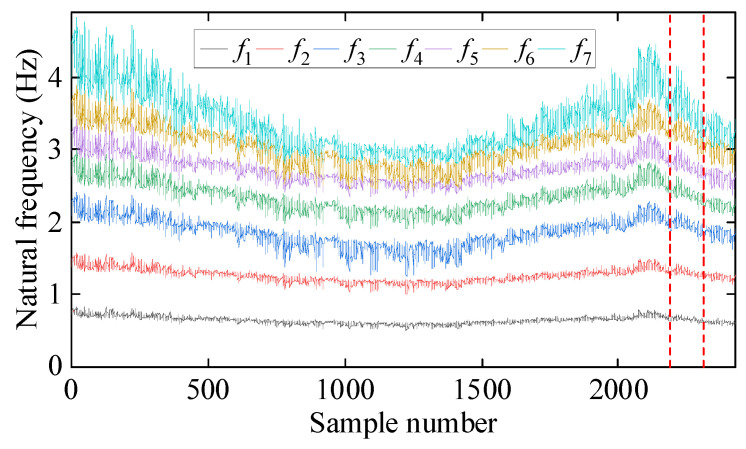
Evolution of the first seven natural frequencies over time.

Figure 4 presents the time series of the seven natural frequencies, with two dashed vertical lines indicating the moments of damage occurrence. It is evident that changing temperature has significant effects on these frequencies, the frequency changes induced by temperature variations exceed those caused by damage. Therefore, relying solely on frequency observations complicates damage detection, necessitating the removal of temperature effects from the frequency data.

### 4.1. Damage Detection

**Figure 5:**
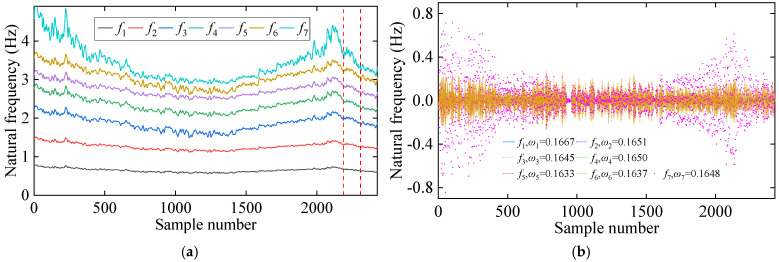
The decomposition results of natural frequencies of the 7-DOF system: (**a**) IMF1, (**b**) IMF2.

**Figure 6:**
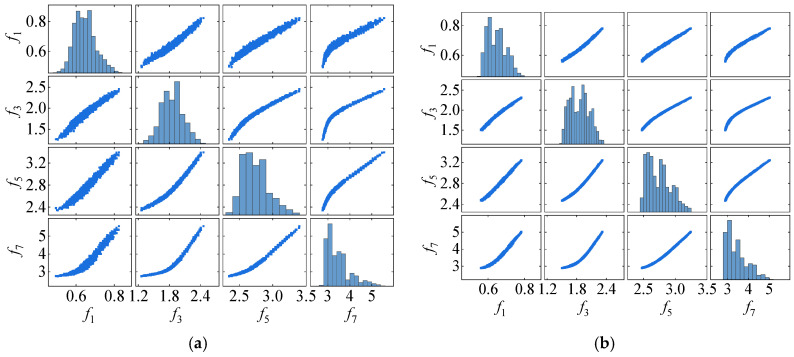
The distribution of interrelationships in frequency signals of the 7-DOF model. (**a**) original frequency. (**b**) IMF1.

The initial step of the proposed method involves using VMD to separate seasonal patterns and noise in the frequency data. Two modes are extracted, IMF1 and IMF2, as depicted in Figure 5. The center frequencies of IMF1 are all zero, while IMF2 has an average center frequency of approximately 0.165 cycles. Because temperature data are collected every four hours, the corresponding frequency data are also sampled at the same interval. This results in IMF2 representing a cyclical pattern, with an average center frequency of around 0.1647 cycles per four hours, close to one full cycle per 24 h. Therefore, IMF2 reflects daily temperature variations, and these signals are removed from the original frequency data. Furthermore, Figure 6a shows the distribution of the original frequency data and the correlations between frequency pairs, while Figure 6b displays the same after the removal of IMF2 signals, leaving only IMF1. As observed, the off-diagonal plots in both figures show the nonlinear relationship between frequency pairs, which is caused by the bilinear interaction between stiffness and temperature. Meanwhile, it can be seen that the original frequency data exhibit non-Gaussian distributions. Although the noise level decreases in the co-distribution plots (off-diagonal plots) after the application of the VMD, the distributions of the frequency signals (main diagonal plots) continue to reveal non-Gaussian distributions. This shows that removing the seasonal pattern does not change the distribution of the data.

**Figure 7:**
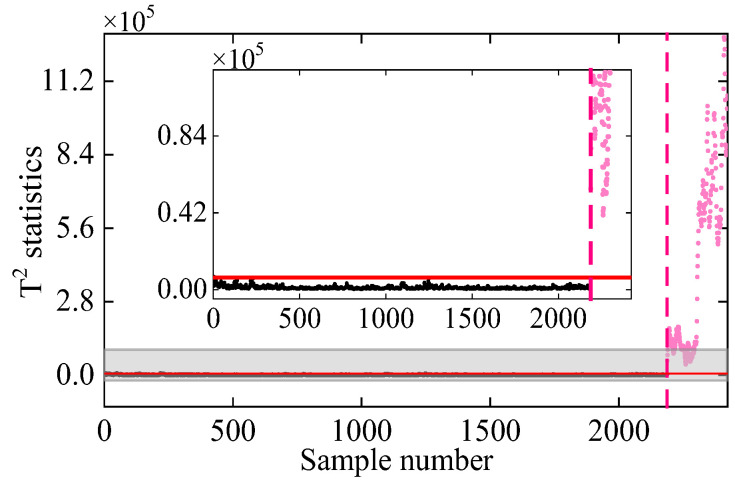
Damage detection result obtained by the proposed VMD-DKECA method. (The horizontal red solid line indicates the threshold, the vertical red dashed line indicates when damage occurs, and the pink dots indicate the $T^{2}$ statistics in the damage state).

Once the seasonal patterns (IMF2) are eliminated, the remaining IMF1 signals are used for damage detection. In this study, IMF1 signals from normal conditions are used as the training dataset, while those from damaged states serve as test data. Following the procedure in Section 3, the damage detection result obtained by the proposed method is shown in Figure 7. To clearly show the sample points of undamaged and damaged states near the threshold, a detailed plot of $T^{2}$ statistics close to the threshold line is also plotted. As observed in the undamaged state, the $T^{2}$ statistics remain below the threshold and stationery, indicating that the nonlinear environmental effects have been effectively filtered out. In the damaged state, $T^{2}$ statistics consistently exceed the threshold, and the values rise with increasing damage severity. This demonstrates the good ability of the proposed method to detect the occurrence of damage for the 7-DOF system.

### 4.2. Comparison of the Proposed Method with Other Methods

Despite the promising damage detection results achieved by the proposed VMD-DKECA method, a comparative study is conducted to further demonstrate its advantages over other state-of-the-art techniques. This study consists of two parts. First, the performance of the VMD-DKECA method is compared with PCA and KPCA to highlight its effectiveness in handling nonlinear and non-Gaussian data. Second, a comparison between the DKECA method and conventional KECA is carried out to showcase the benefits of considering dynamic data.

**Figure 8:**
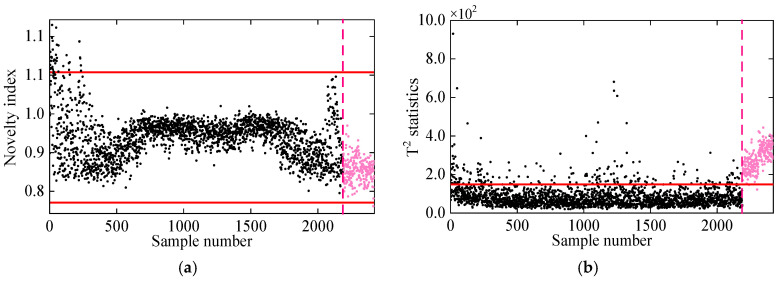
Damage detection results obtained by the (**a**) PCA and (**b**) KPCA methods. (The horizontal red solid line indicates the threshold, the vertical red dashed line indicates when damage occurs, and the pink dots indicate the $T^{2}$ statistics in the damage state).

In the first comparative analysis, Figure 8 presents the damage detection results for PCA and KPCA. As shown, the PCA method fails to reliably detect damage, as its novelty indices remain within the control limits even under damaged conditions. This is primarily due to the linear nature of PCA, which limits its effectiveness in handling the nonlinearly related frequency data of the 7-DOF system. On the other hand, KPCA, which utilizes the kernel trick to handle nonlinear data, performs better in identifying damage. However, some residuals in the undamaged state exceed the control limits, leading to false detections. This is likely due to KPCA’s assumption of Gaussian data distribution, while the frequencies in the 7-DOF system exhibit non-Gaussian characteristics, reducing its damage detection accuracy.

**Figure 9:**
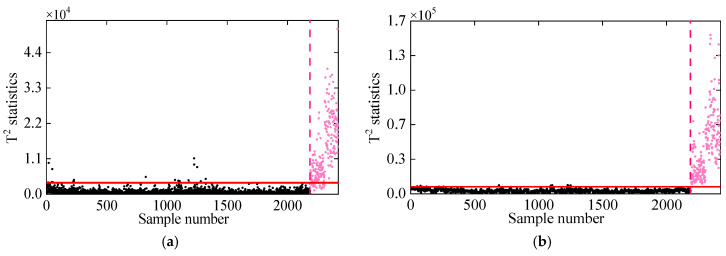
Damage detection results obtained by the (**a**) KECA and (**b**) DKECA methods. (The horizontal red solid line indicates the threshold, the vertical red dashed line indicates when damage occurs, and the pink dots indicate the $T^{2}$ statistics in the damage state).

In the second comparative study, DKECA is compared with KECA. Figure 9 illustrates the damage detection results for both methods applied to the 7-DOF system. The results indicate that both KECA and DKECA outperform KPCA, as they are not restricted by data distribution assumptions. However, some $T^{2}$ statistics from KECA in the undamaged state exceed the threshold, while others in the damaged state fall below it, leading to false alarms and missed detections. In contrast, DKECA performs significantly better, accurately detecting damage without false alarms or missed detections. These results confirm that the inclusion of time-lagged data in DKECA enhances the available information by capturing correlations in the dynamic frequency data, improving its overall detection accuracy.

To further evaluate the accuracy of various damage detection methods, two primary metrics—false positive rate (FPR) and false negative rate (FNR)—are applied. These metrics, commonly employed in machine learning, are derived from the confusion matrix, shown in Table 1. This matrix organizes the algorithm’s performance based on true positives (TPs), false positives (FPs), false negatives (FNs), and true negatives (TNs). In the context of SHM, a “positive” outcome indicates a damaged state, while a “negative” outcome signifies an undamaged state. True negatives occur when the structure is undamaged, and the method correctly identifies it as such. False negatives arise when the structure is damaged, yet the method fails to detect it. Conversely, false positives happen when the structure is undamaged, but the method incorrectly identifies it as damaged. True positives signify that the structure is damaged, and the method correctly detects this.

(17) $$FPR=\frac{N_{FP}}{N_{FP}+N_{TN}}\times 100\%$$

(18) $$FNR=\frac{N_{FN}}{N_{FN}+N_{TP}}\times 100\%$$

where $N_{TN},\;N_{FN},\;N_{FP},$ and $N_{TP}$ represent the number of *TN*, *FN*, *FP* and *TP* sample points.

Table 2 outlines the FPR and FNR for each method evaluated. The results show that PCA has the worst performance due to its high FNR, indicating a substantial misclassification of damaged states as normal. Although KPCA improves the FNR compared to PCA, it still exhibits a high FPR, which reflects its overall poor performance. This is primarily due to KPCA’s assumption of Gaussian data distribution, limiting its effectiveness with non-Gaussian monitoring data. Traditional PCA and KPCA methods identify principal components by maximizing the variance in the data. They assume that the data’s variability is the most informative feature. However, when data are non-Gaussian, especially if they have skewed or heavy-tailed distributions, variance may not adequately capture the underlying structure. In such cases, PCA might not effectively reveal the true patterns in the data. In contrast, the KECA, DKECA, and VMD-DKECA methods leverage entropy, a fundamental concept in information theory, to analyze the distribution of data. By focusing on entropy and utilizing kernel methods, the KECA, DKECA, and VMD-DKECA methods do not rely solely on second-order statistics like covariance, which can be misleading for non-Gaussian data. Instead, the entropy-based methods capture higher-order statistical properties, providing a more comprehensive analysis of the data’s structure. Therefore, KECA addresses this limitation by removing the Gaussian assumption, resulting in more accurate damage detection. Furthermore, the VMD-DKECA and DKECA methods outperform all other techniques in terms of overall performance, due to their ability to account for time correlations in dynamic frequency data. This confirms the robustness of the proposed approach in detecting structural damage and mitigating the effects of nonlinear environmental variability.

### 4.3. Effect of Measurement Noise, Data Nonlinearity Degree, and Frequency Grouping

**Figure 10:**
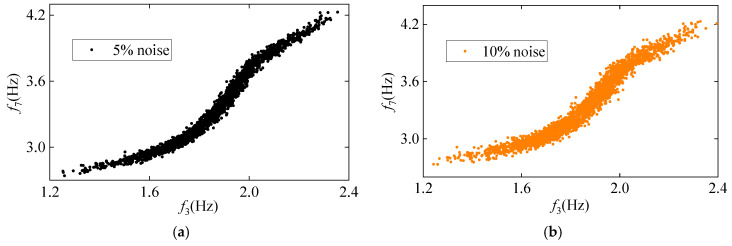
The correlation between $f_{3}$ and $f_{7}$ at the noise levels of (**a**) 5% and (**b**) 10%.

**Figure 11:**
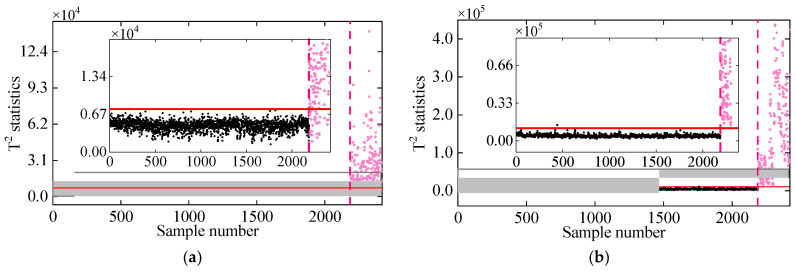
Damage detection results obtained by the (**a**) DKECA and (**b**) VMD-DKECA methods at the noise level of 5%. (The horizontal red solid line indicates the threshold, the vertical red dashed line indicates when damage occurs, and the pink dots indicate the $T^{2}$ statistics in the damage state).

**Figure 12:**
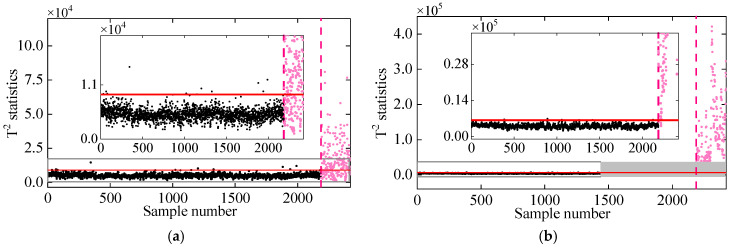
Damage detection results obtained by the (**a**) DKECA and (**b**) VMD-DKECA methods at the noise level of 10%. (The horizontal red solid line indicates the threshold, the vertical red dashed line indicates when damage occurs, and the pink dots indicate the $T^{2}$ statistics in the damage state).

To assess the impact of measurement noise on the proposed method’s performance, frequency data with Gaussian white noise levels of 5% and 10% were employed for damage detection. Figure 10 shows the correlation between frequencies $f_{3}$ and $f_{7}$ at noise levels of 5% and 10%. As can be seen, the correlation diminishes as the noise level increases from 5% to 10%, indicating that measurement noise adversely affects frequency correlation. Moreover, Figure 11 and Figure 12 show the damage detection results of the proposed VMD-DKECA method as well as the DKECA method under different noises. It can be seen that, in the presence of measurement noise, the detection result of this method is more accurate than that of the DKECA method. This indicate that the noise seriously affects the performance of the DKECA method and that reliable damage detection can be achieved by removing these effects using VMD. Although the detection accuracy of this method slightly decreases as the noise increases, the method is capable of reliably distinguishing between damaged and undamaged states even in the presence of high measurement noise.

As described in the section above, the proposed method is a nonlinear method for separating environmental impacts and can be applied to situations where nonlinear relationships exist between frequencies. To investigate the effect of nonlinear intensity on the performance of the proposed method, an analysis was conducted on different nonlinearly correlated data. The nonlinear intensity of the data is determined by the variations in values $a_{1}$ and $a_{2},$ as described in Equation (19). When $a_{1}$ and $a_{2}$ are equal, the relationship between the damage features is linearly correlated [42]. As the difference between $a_{1}$ and $a_{2}$ increases, it is assumed that the nonlinear intensity increases. Four nonlinear relationships are considered to examine the ability of the proposed method for damage detection of data with different nonlinear correlations, as shown in Table 3.

(19) $$k_{i}=\left\{\begin{array}{c} a_{1}\times T+b_{1},\;if\;T<0\\ a_{2}\times T+b_{2},\;if\;T\geq 0 \end{array}\right.,i=1,\ldots ,8$$

**Figure 13:**
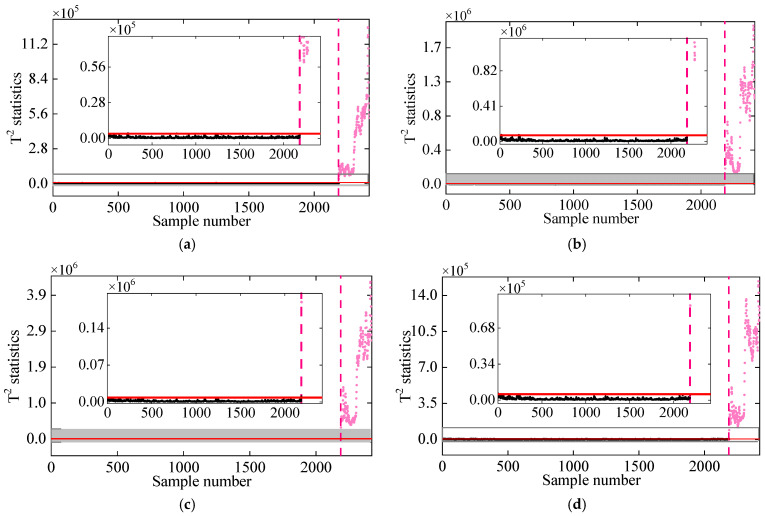
Damage detection by the proposed method for different nonlinear cases: (**a**) Case 1, (**b**) Case 2, (**c**) Case 3, and (**d**) Case 4. (The horizontal red solid line indicates the threshold, the vertical red dashed line indicates when damage occurs, and the pink dots indicate the $T^{2}$ statistics in the damage state).

Figure 13 shows the performance of the proposed method in different nonlinear cases. For all nonlinear cases, the $T^{2}$ statistics remains stationary and below the threshold in the undamaged state, but it significantly jumps and exceeds the UCL line once damage occurs. Moreover, even if the monitoring data exhibit a high degree of nonlinearity, it can be perceived that there is an obvious difference in the $T^{2}$ statistics of the normal and damaged conditions. These conclusions eventually confirm the effectiveness of the proposed method in damage detection of the 7-DOF system under different nonlinear cases and also prove its high damage detectability.

**Figure 14:**
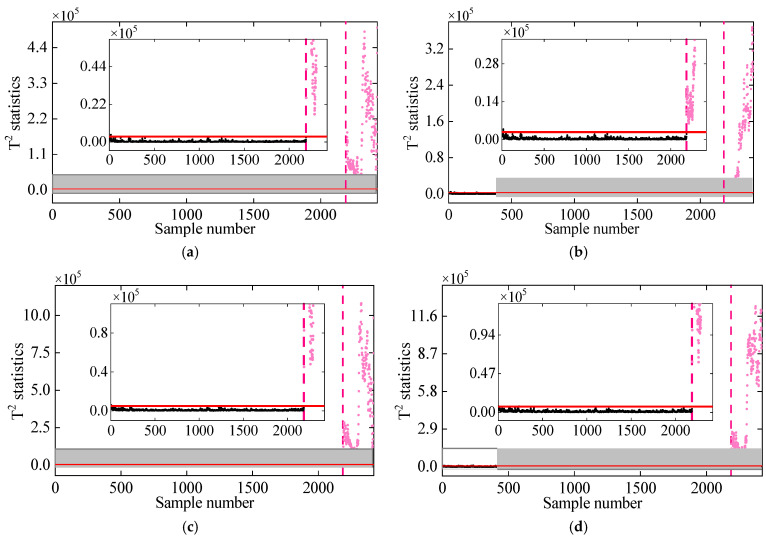
Damage detection results obtained by the proposed method for different frequency groupings: (**a**) $\left[f_{3},f_{4}\right]$, (**b**) $\left[f_{1},f_{3},f_{5}\right]$, (**c**) $\left[f_{1},f_{2},f_{3},f_{4}\right],$ and (**d**) $[f_{1},f_{2},f_{3},f_{4},f_{5}].$ (The horizontal red solid line indicates the threshold, the vertical red dashed line indicates when damage occurs, and the pink dots indicate the $T^{2}$ statistics in the damage state).

Due to challenges in accurately identifying a sufficient number of modal parameters, particularly higher-order modes, the impact of the number of selected frequencies on the performance of the proposed detection method is examined. Figure 14 illustrates the damage detection results obtained using varying numbers of frequencies. In Figure 14, it can be observed that the $T^{2}$ statistics for the undamaged state remain stable and consistently below the threshold, indicating that the normal condition is detected accurately without any false alarms. Additionally, the $T^{2}$ statistics for test samples in the damaged condition consistently exceed the threshold, with an increase in $T^{2}$ values corresponding to greater damage intensity. This suggests that the method effectively detects damage as it occurs. Furthermore, regardless of the threshold, a clear distinction can be observed between the high quantiles of the normal and damaged conditions. These findings confirm the effectiveness of the proposed method in detecting damage in the 7-DOF structure, even with a limited amount of frequency data.

## 5. An Application to the Z24 Bridge

Experimental data from the Z24 bridge are utilized in this study to further validate the proposed method. The Z24 bridge is recognized within the structural health monitoring community as a benchmark for damage detection under nonlinear environmental effects. Located in Switzerland, this three-span pre-stressed highway bridge was constructed between 1961 and 1963 to connect the villages of Koppigen and Utzenstorf. Although the bridge remained operational, it was demolished at the end of 1998 to accommodate a new bridge. Prior to its demolition, the bridge was monitored for nearly a year by the De Roeck G team, which collected data on environmental variability, including temperature and humidity, as well as dynamic responses such as acceleration [9]. Additionally, progressive damage tests were performed shortly before demolition to gather realistic damage data for the structure.

**Figure 15:**
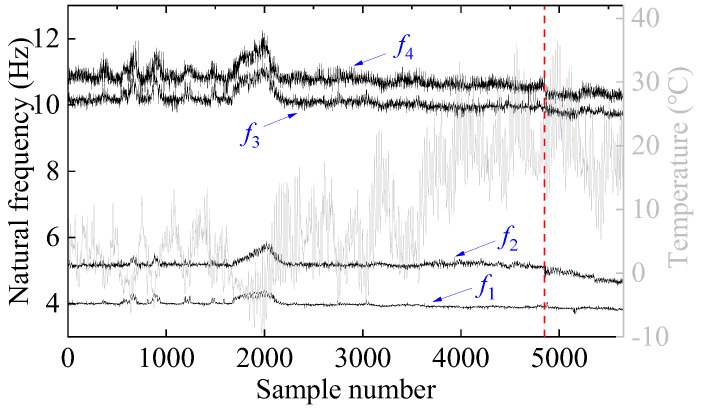
Evolution of air temperature and first four natural frequencies of Z24 bridge over time. (The vertical red dashed line indicates when damage occurs).

The long-term dynamic characteristics of the Z24 bridge were identified in four modes using the stochastic subspace method. Figure 15 presents the variations in air temperature alongside the series of identified natural frequencies over time, with the onset of damage marked by a vertical dashed line. It is apparent that changing environmental temperature causes substantial amplitude changes in frequency; these changes far exceed the relative frequency variations observed in the damaged state. Such fluctuations may lead to incorrect damage detection, underscoring the importance of removing environmental influences from frequency data.

**Figure 16:**
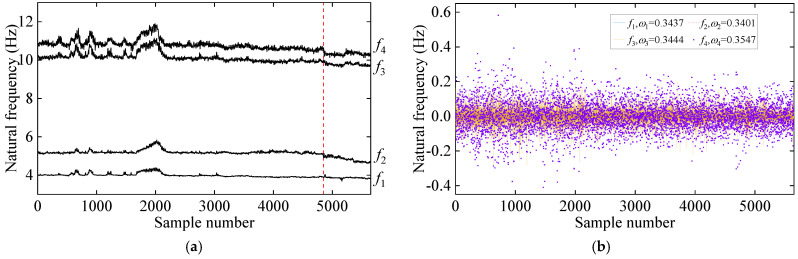
The decomposition results of the natural frequency via VMD: (**a**) IMF1 and (**b**) IMF2. (The vertical red dashed line indicates when damage occurs).

**Figure 17:**
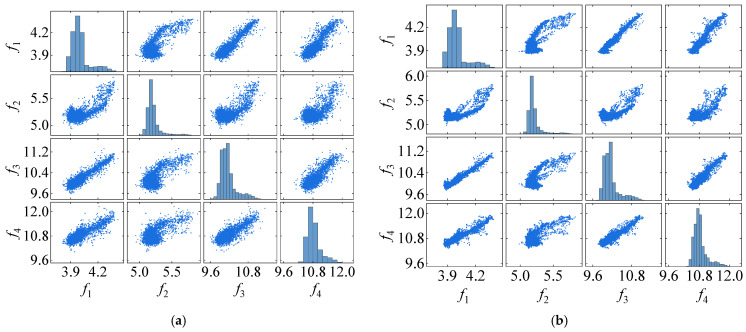
The distribution of interrelationships in frequency signals of the Z24 bridge. (**a**) Original frequency and (**b**) IMF1.

Following the process of the proposed damage detection method, the natural frequency time series is first decomposed into two IMFs using the VMD method. The results of this decomposition, along with their center frequencies, are presented in Figure 16. Notably, the center frequency of IMF1 signals is zero. In contrast, the average center frequency of IMF2 signals is approximately 0.346, reflecting seasonal environmental effects. Consequently, IMF2 signals are excluded, and only IMF1 signals are employed for damage detection. Figure 17a illustrates the distribution and mutual co-distribution of the modal frequencies of the Z24 bridge, while Figure 17b illustrates the distribution and mutual co-distribution of the IMF1s. Similar to the observations made in the 7-DOF numerical example, nonlinear relationships between the modal frequencies are evident, particularly a bilinear relationship between $f_{2}$ and other frequencies. Furthermore, the main diagonal entries reveal that the frequency distribution is non-Gaussian, highlighting the complexity of the Z24 frequency data.

**Figure 18:**
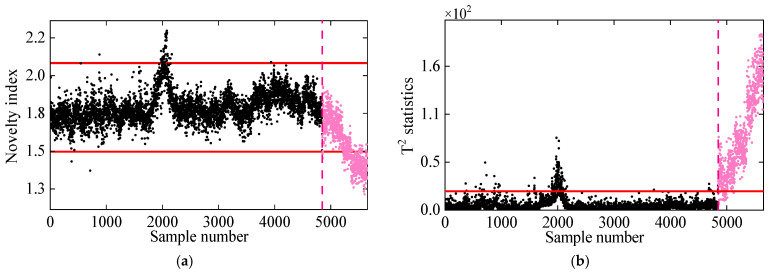

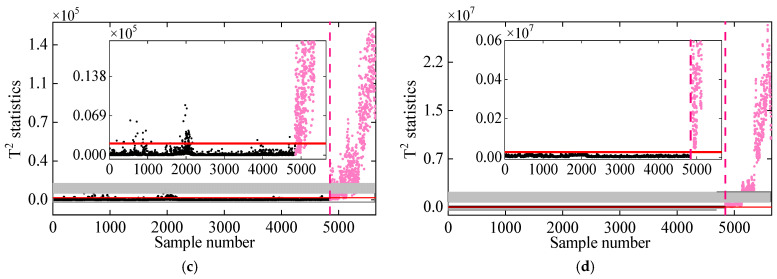
Damage detection results obtained by the (**a**) PCA, (**b**) KPCA, (**c**) KECA, and (**d**) VMD-DKECA methods. (The horizontal red solid line indicates the threshold, the vertical red dashed line indicates when damage occurs, and the pink dots indicate the $T^{2}$ statistics in the damage state).

To assess the proposed method’s effectiveness, a comparative analysis is conducted against some state-of-the-art techniques. As illustrated in Figure 18, the PCA, KPCA, KECA, and VMD-DKECA methods display distinct damage detection results. Table 4 further provides the corresponding FPR and FNR for each method. As can be seen, the best performance belongs to the proposed VMD-DKECA method. In contrast, the worst performance is related to the PCA-based technique. This discrepancy is largely attributed to PCA’s linear nature, which limits its ability to adequately filter nonlinear environmental influences from frequency data. Although the KPCA and KECA methods perform better than PCA in detecting the damaged state, the proposed method yields a more reliable result compared to these techniques. These findings confirm the proposed algorithm’s positive effect in mitigating the environmental variability condition and validate its enhanced capacity to manage dynamic, nonlinear, and non-Gaussian frequency data effectively.

The comparison of calculation times for different methods applied to the Z24 bridge is shown in Table 5. The calculations were performed on an Intel i7 CPU with a 2.5 GHz clock speed and 16 GB of RAM running MATLAB R2019a. It can be observed that kernel-based methods (KPCA, KECA, DKECA) require more computation time than the PCA method, which lacks a kernel. This is due to the kernel method mapping vectors from a low-dimensional space to a higher-dimensional one, thus increasing the matrix dimension and computational cost. However, despite the involvement of additional matrix operations and eigenvalue decompositions in the proposed VMD-DKECA method, the increase in computational cost is minimal. This indicates that the proposed method maintains good computational efficiency.

**Figure 19:**
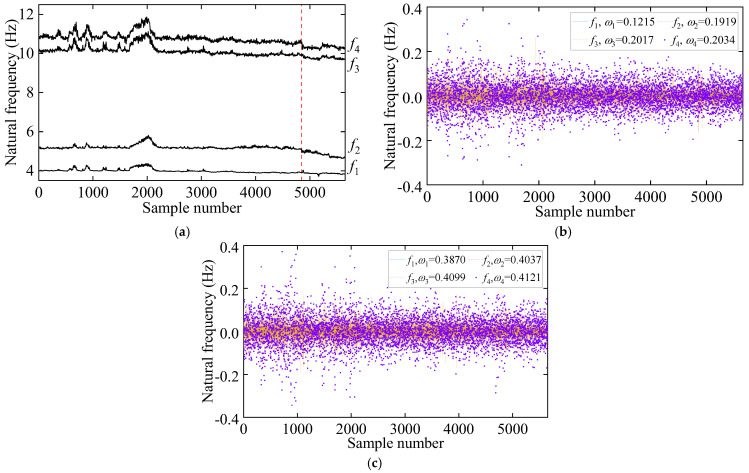
The decomposition results of the natural frequency for Z24 bridge via VMD: (**a**) IMF1, (**b**) IMF2, and (**c**) IMF3. (The vertical red dashed line indicates when damage occurs).

**Figure 20:**
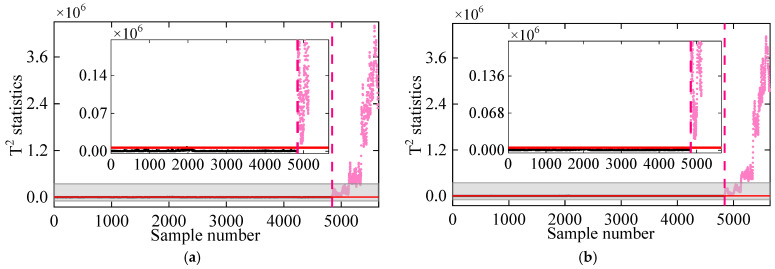
Damage detection results obtained by the proposed method when the frequency signals are decomposed into (**a**) three and (**b**) four IMFs. (The horizontal red solid line indicates the threshold, the vertical red dashed line indicates when damage occurs, and the pink dots indicate the $T^{2}$ statistics in the damage state).

In this paper, the primary role of VMD is to eliminate seasonal patterns and noise from the original frequency signal. To assess the effect of the number of decomposed IMFs on the proposed method, the frequency signal is decomposed into three and four IMFs, respectively. IMFs with periodic patterns are discarded, leaving only IMF1, which has a center frequency of 0, for damage detection. Figure 19 shows the decomposition results when the frequency signals are decomposed into three IMFs. Figure 20 illustrates the damage detection results when the IMF1s are used from decomposed three and four IMFs, respectively. In both instances, accurate damage detection is achieved, suggesting that the number of decomposed IMFs has minimal impact on the method’s performance. This is primarily because, regardless of the number of decompositions, the long-term nonstationary pattern is preserved, while seasonal patterns and noise are removed. This supports the development of a precise Damage Identification Model based on the DKECA method. Consequently, for simplicity, it is recommended to decompose the frequency signal into only two IMFs, excluding IMF2, which contains the periodic pattern, to minimize the influence of seasonal environmental factors.

**Figure 21:**
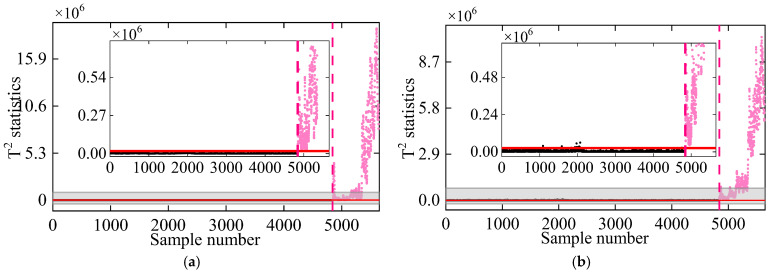
Damage detection results obtained by (**a**) the EMD-DKECA method and (**b**) the WA-DKECA method. (The horizontal red solid line indicates the threshold, the vertical red dashed line indicates when damage occurs, and the pink dots indicate the $T^{2}$ statistics in the damage state).

Moreover, we have included empirical mode decomposition (EMD) and wavelet analysis (WA) as alternative preprocessing tools for damage detection. Firstly, the frequency signal is decomposed into multiple IMFs based on the EMD method. After the periodic components are removed, the remaining IMF is utilized for damage detection, with the results displayed in Figure 21a. A similar approach is applied using wavelet decomposition to eliminate the periodic components in the signal, followed by the DKECA method for damage identification, as shown in Figure 21b. The results from both methods demonstrate high accuracy in damage detection. This indicates that strict requirements for the choice of preprocessing tools are unnecessary. As long as the periodic components are successfully removed from the original frequency data, reliable damage identification can be achieved.

**Figure 22:**
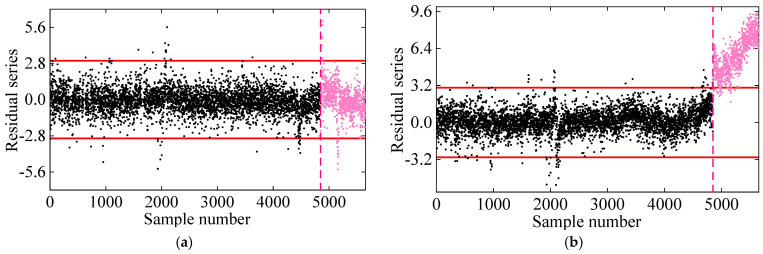
Damage detection results obtained by the regime-switching cointegration method using (**a**) −0.77 °C and (**b**) 0.615 °C as the temperature breakpoints [23]. (The horizontal red solid line indicates the threshold, the vertical red dashed line indicates when damage occurs, and the pink dots indicate the $T^{2}$ statistics in the damage state).

To address the nonlinear environmental effects and detect damage in the Z24 bridge, a regime-switching cointegration method is introduced in [23]. This method extends the conventional Johansen cointegration technique into a nonlinear context, enabling the establishment of a switching cointegration relationship between damage features through a switching point. By applying this regime-switching approach using different temperature breakpoints, damage detection results can be obtained, as illustrated in Figure 22. The results show that, when −0.77 °C is chosen as the switching point, structural damage cannot be effectively identified. However, selecting 0.615 °C as the switching point allows for accurate detection of damage. This highlights the importance of the temperature-switching point in influencing the performance of the regime-switching cointegration method. In contrast, the VMD-DKECA algorithm proposed here utilizes a kernel method to address the nonlinear problem, offering precise damage detection without the need for a switching point.

**Figure 23:**
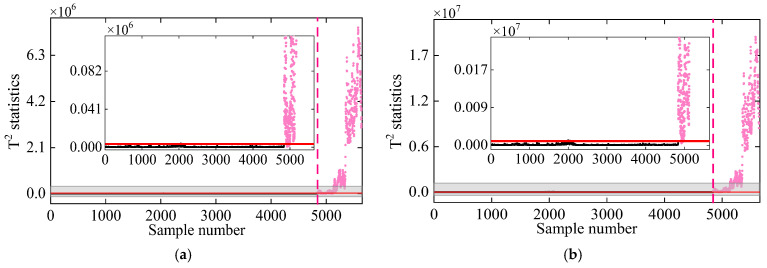
Damage detection results obtained by the proposed method for different frequency groupings: (**a**) $\left[f_{1},f_{2}\right]$ and (**b**) $[f_{1},f_{2},f_{3}].$ (The horizontal red solid line indicates the threshold, the vertical red dashed line indicates when damage occurs, and the pink dots indicate the $T^{2}$ statistics in the damage state).

Similar to the 7-DOF numerical example, the performance of the proposed method was also evaluated using different numbers of frequencies. Figure 23 shows the damage detection results with two frequencies $\left[f_{1},f_{2}\right]$ and three frequencies $\left[f_{1},f_{2},f_{3}\right]$. The method successfully identified damage in both cases, with $T^{2}$ statistics increasing as the severity of the damage grew. Even with just two frequencies, the method could distinguish between undamaged and damaged states. This confirms that the proposed method has excellent damage detection performance, even with limited frequency data.

**Figure 24:**
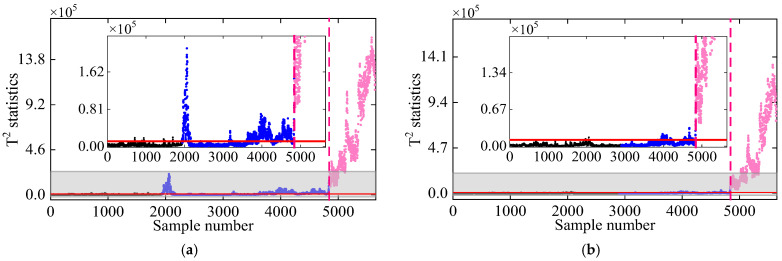

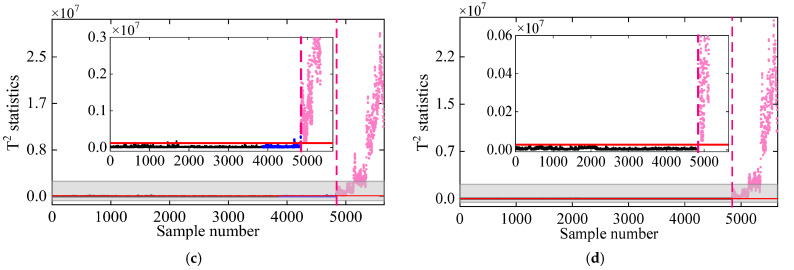
Damage detection in the Z24 Bridge based on the proposed method using (**a**) 40%, (**b**) 60% (**c**) 80%, and (**d**) 100% of the health data as the training set, respectively. (The horizontal red solid line indicates the threshold, the vertical red dashed line indicates when damage occurs, and the pink dots indicate the $T^{2}$ statistics in the damage state).

In previous analyses, 100% of the natural frequency observations under normal conditions were used as training data for damage detection. To assess the impact of training dataset size on the method’s performance, additional datasets were created using 40%, 60%, and 80% of the undamaged frequency observations. Following the procedure outlined in Section 3, the $T^{2}$ statistics were calculated for both the training and testing stages, as depicted in Figure 24. When using 40% of the undamaged frequencies as training data, a spike in $T^{2}$ statistics appears around the 2000th sample in the undamaged stage, indicating incorrect damage identification. However, as the training dataset size increased to 60%, the false positive rate in the undamaged state reduced significantly. With 80% of undamaged frequencies as training data, $T^{2}$ statistics for the undamaged stage consistently remained below the threshold, while those for the damaged condition surpassed it, effectively distinguishing the two states. The primary reason for the poor performance with 40% training data is the insufficient representation of frequency data under low-temperature conditions, leading to an inaccurate DKECA model. This highlights the importance of using a sufficiently large and diverse training dataset that encompasses a wide range of environmental variations to ensure reliable and accurate damage detection.

## 6. Conclusions

This paper introduces a VMD-DKECA method for damage detection in SHM under varying environmental conditions. The method involves three key steps: data pre-processing using VMD, constructing the DKECA model, and detecting damage through $T^{2}$ monitoring statistics. In the first step, VMD is employed to reduce noise in frequency data. The resulting IMF1s from VMD are used to create a time-lagged matrix, which serves as the basis for constructing the DKECA model. This model is then used to develop a damage detector based on $T^{2}$ statistics. The proposed approach was initially tested on a numerical example and later validated with long-term measurements from the Z24 bridge. Key conclusions are as follows:

(1) The proposed method, in conjunction with the VMD and DKECA, could significantly mitigate the influences of the environmental variations, accurately identifying damage in both tested structures. Comparative studies indicate that this method outperforms traditional techniques, such as PCA, KPCA, and KECA, particularly in handling dynamic, nonlinear, and non-Gaussian data.
(2) VMD plays a critical role in the success of this method by filtering out measurement noise. The VMD-DKECA algorithm was shown to be more effective than the DKECA method alone when dealing with noisy frequency data.
(3) The proposed VMD-DKECA method demonstrates high accuracy in detecting damage, even with limited frequency data and strong data nonlinearity. However, the performance depends on the size of the training dataset. A sufficiently large and varied dataset that captures a broad range of environmental conditions is essential for reliable damage detection.

## Figures and Tables

**Table 1:** The confusion matrix for evaluating the performance of damage detection method.

Detected Results	Actual State	
	Undamaged	Damage
Undamaged	TN	FN
Damage	FP	TP

**Table 2:** FPR and FNR obtained based on different damage detection methods in the 7-DOF example.

	PCA	KPCA	KECA	DKECA	VMD-DKECA
FPR	1.69%	9.36%	0.90%	0.10%	0.04%
FNR	99.58%	0.84%	10.50%	0.03%	0%

**Table 3:** Simulate different degrees of nonlinear relationships by setting different values of $a_{1}$ and $a_{2}$.

	$k_{3}$	$k_{1},k_{2},k_{4},k_{5},k_{6},k_{7},k_{8}$
Case 1	$a_{1}=-0.75,a_{2}=-0.25,b_{1}=10,b_{2}=10$	$a_{1}=-0.15,a_{2}=-0.15,$ $b_{1}=6,b_{2}=6$
Case 2	$a_{1}=-1.00,a_{2}=-0.25,b_{1}=10,b_{2}=10$	
Case 3	$a_{1}=-1.25,a_{2}=-0.25,b_{1}=10,b_{2}=10$	
Case 4	$a_{1}=-1.50,a_{2}=-0.25,b_{1}=10,b_{2}=10$	

**Table 4:** FPR and FNR obtained based on different damage detection methods in the Z24 bridge.

	PCA	KPCA	KECA	VMD-DKECA
FPR	0.31%	10.83%	1.18%	0.07%
FNR	67.6%	0.12%	4.87%	0%

**Table 5:** Comparison of computational costs for different methods.

	PCA	KPCA	KECA	VMD-DKECA
Computational cost (s)	1.1	5.2	4.2	4.8

## Data Availability

The research data will be available from the corresponding author upon reasonable request.
